# Pressure Anaesthesia

**Published:** 1904-06

**Authors:** J. J. E. De Vries

**Affiliations:** Amsterdam, Holland.


### PRESSURE ANAESTHESIA.

By DR. J. J. E. DE VRIES, Amsterdam, Holland.

I have used pressure anæsthesia in connection with cocaine for over four years, and am really very much pleased with the results. The hemorrhage after extirpation was the only unpleasant feature, often requiring much time to be controlled. Since November, 1902, I have used cocaine and adrenalin, changing several times the formula. For about six months I have relied on the following solution, which, if kept in a dark, well closed glass stoppered bottle, keeps all right for months:

Sol. Adrenalin ....................

1-1000—5. Parke Davis

Hydro Chl. Cocaine.............

1.

My *modus operandi* is as follows: Syringe cavity several times with a weak Lysol solution (1-1000). Put on rubber dam directly. Wash out cavity with Lysol, dry with cotton and place in the cavity a small piece of cotton with adrenalin-cocaine, leaving it there for from three to five minutes. Excavating with no pain at all is possible, and where not exposed, expose the pulp quickly with the aid of a sharp fissure bur. Wash out cavity with absolute alcohol (less irritant than formalin, etc.). Apply again cocaine-adrenalin; put a piece of unvulcanized rubber and under slowly increasing pressure impregnate the whole pulp. Open pulp chamber widely and extirpate immediately. Wash out canals with thirty per cent. $H^2 O^2$, followed by absolute alcohol and dry with hot air. Except in very few cases I fill canals immediately as follows:

Wash out canals with cotton twisted around a Miller's needle, dipped first in a saturate solution of thymol in oil of cinnamon; fill with G.P. points dipped in the same solution. The oil of cinnamon being evaporated after some time, leaves the thymol in small crystals along the wall of the root canal and with the G.P. points form in this way a very long lasting antiseptic. Where necessary (mesial root lower molar, etc.) I enlarge the canals with the aid of Beutebrock's instrument, preferring the hand burs. I have followed this way of treatment daily and thus far have had no bad after effects. At the December meeting of the National Dental Association I gave a lecture on direct pulp extirpation with cocaine-adrenalin, followed by a demonstration. Many dentists are now practicing this mode of extirpation, all being very much pleased with the success.

